# Supplementary material for: Speckles and paraspeckles coordinate to regulate HSV-1 genes transcription
Source: Commun Biol. 2021 Oct 21;4:1207. doi: 10.1038/s42003-021-02742-6 (PMC8531360; doi:10.1038/s42003-021-02742-6)
Supplement: Supplementary file 2 — Supplementary Information [file 42003_2021_2742_MOESM2_ESM.pdf]

Supplementary Materials

Supplementary Figures

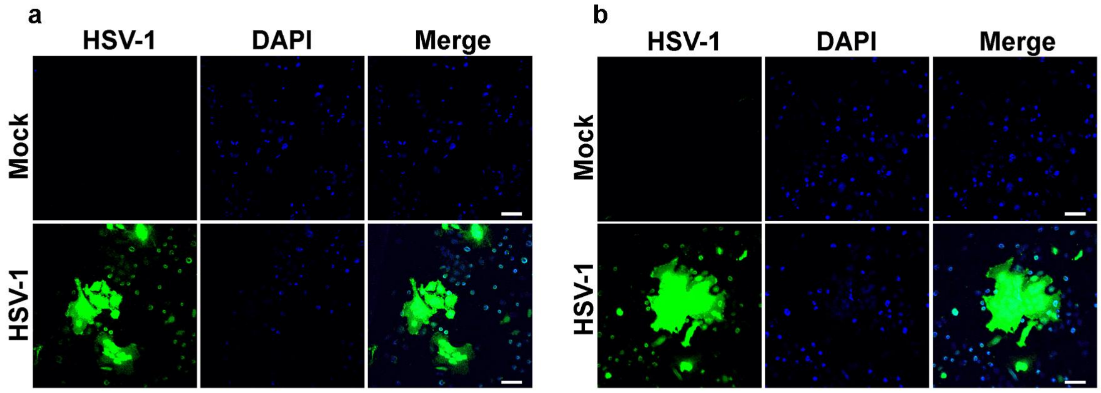

Supplementary Figure 1. The infection efficiency of HSV-1 in HeLa cells and C-33A cells. a, **b** HeLa cells (**a**) or C-33A cells (**b**) infected with HSV-1 or Mock for 12 hours were fixed and immuno-stained with an anti-HSV-1 glycoprotein antibodies (green). Images were captured with a confocal microscope. Nuclei were stained with DAPI (blue). Scale bars, 80 µm.

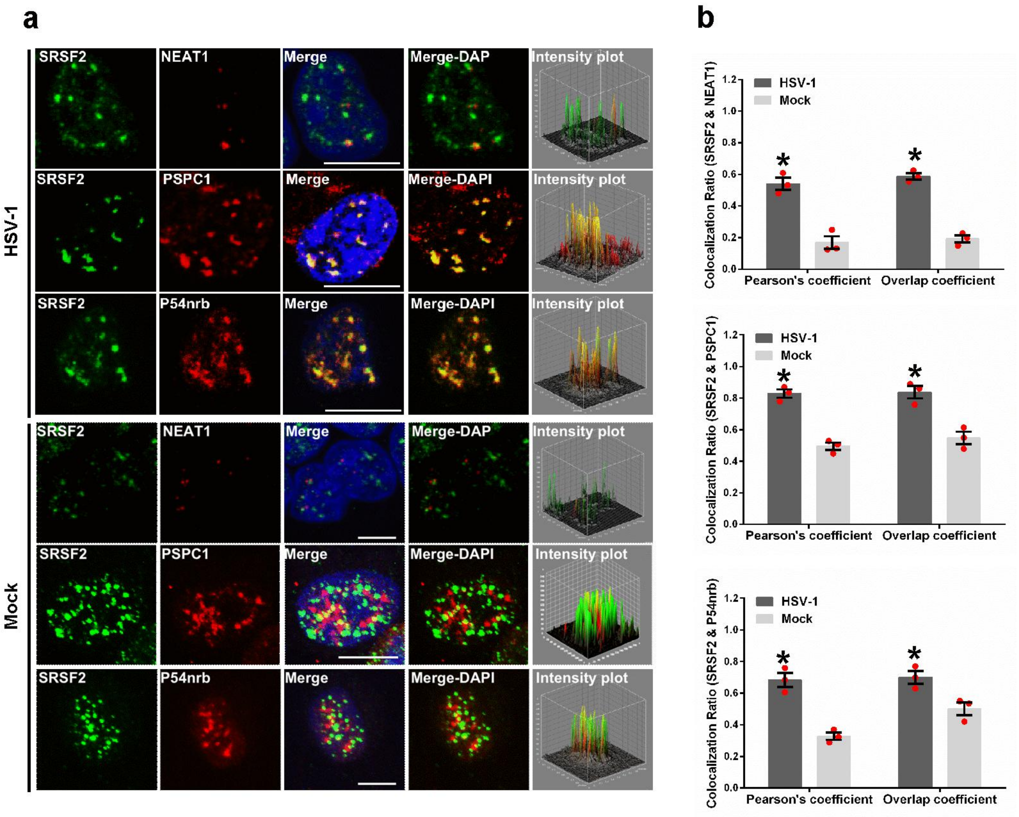

Supplementary Figure 2. HSV-1 infection redistributed speckles and paraspeckles in C-33A cells. **a** C-33A cells infected with HSV-1 or Mock for 4 hours were incubated with anti-SRSF2 antibodies (green), and then incubated with *NEAT1* probe (red), anti-PSPC1 antibodies (red) or anti-P54nrb antibodies (red). The images were captured with a confocal microscope. The intensity plots for the red and green channels were analyzed by ImageJ software. DAPI (blue) was used to stain the nuclei. Scale bars, 10 μm. **b** The Pearson's coefficient and overlap coefficient for each merge channel in Figure S2a were quantified using the JACoP in ImageJ. The data are presented as the mean ± SD from three independent experiments (*$p < 0.01$, Student's *t*-test).

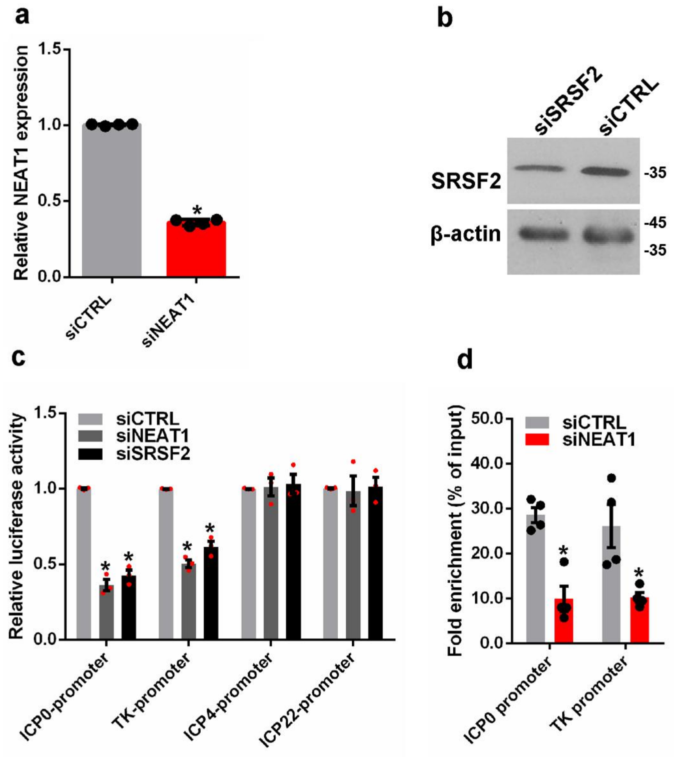

Supplementary Figure 3. ***NEAT1*** **influences the binding ability of SRSF2 to viral genes in C-33A cells. a** C-33A cells transfected with *NEAT1*-targeting siRNAs or negative control siRNAs were infected with HSV-1 for 4 hours. Relative *NEAT1* level compared with *β-actin* mRNA were analyzed with real-time PCR. The data are presented as the mean ± SD from four independent experiments (*p < 0.01, Student's *t*-test). **b** C-33A cells transfected with the SRSF2-targeting siRNAs or negative control siRNAs were infected with HSV-1 for 4 hours. The SRSF2 expression levels were measured with western blotting. **c** After co-transfection with *NEAT1*-targeting siRNAs, SRSF2-targeting siRNAs or negative control and the pGL3 enhancer plasmid containing the *ICP0* gene promoter, *ICP4* promoter, *ICP22* promoter, or a pRL-TK reporter, the C-33A cells were infected with HSV-1 for 4 hours. The relative transcriptional activities were analyzed by luciferase assay. The data are presented as the mean ± SD from three independent experiments (*p < 0.01, Student's *t*-test). **d** C-33A cells transfected with *NEAT1*-targeting siRNAs or negative control siRNAs were infected with HSV-1 for 4 hours. ChIP assays were performed with anti-SRSF2 antibodies, and the fold enrichment of the *ICP0* and *TK* gene promoters by SRSF2 relative to the input level was examined with real-time PCR. The data are presented as the mean ± SD from four independent experiments (*p < 0.01, Student's *t*-test).

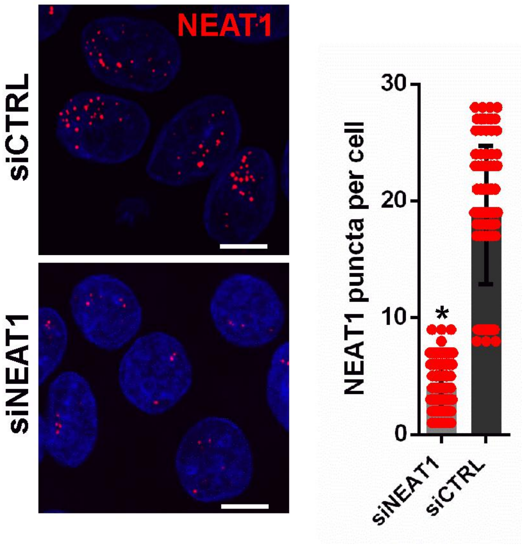

Supplementary Figure 4. The transfection efficiency of *NEAT1*-targeting siRNAs. HeLa cells transfected with *NEAT1*-targeting siRNAs or negative control siRNAs were infected with HSV-1 for 4 hours and then incubated with the *NEAT1* probe (red) overnight. Fluorescence images were captured with a confocal microscope. DAPI (blue) was used to stain the nuclei. Scale bars, 10 µm. The number of *NEAT1* puncta per cell was analyzed (right panels) (*p < 0.01, Student's *t*-test).

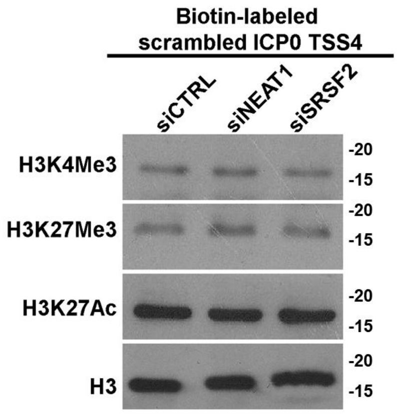

Supplementary Figure 5. ***NEAT1*** **and SRSF2 influence histone modifications located near viral genes.** HeLa cells transfected with *NEAT1*-targeting siRNAs, SRSF2-targeting siRNAs or negative control siRNAs were infected with HSV-1 for 4 hours. Cell lysates were then harvested and incubated with a PCR amplified biotin-labelled scrambled sequence of HSV-1 *ICP0* TSS4. The DNA-protein complexes were pulled down by streptavidin beads and bound proteins were resolved through SDS-PAGE and subsequently analyzed by western blotting assay with anti-Histone H3 antibodies, anti-H3K4Me3 antibodies, H3K27Me3 antibodies or H3K27Ac antibodies.

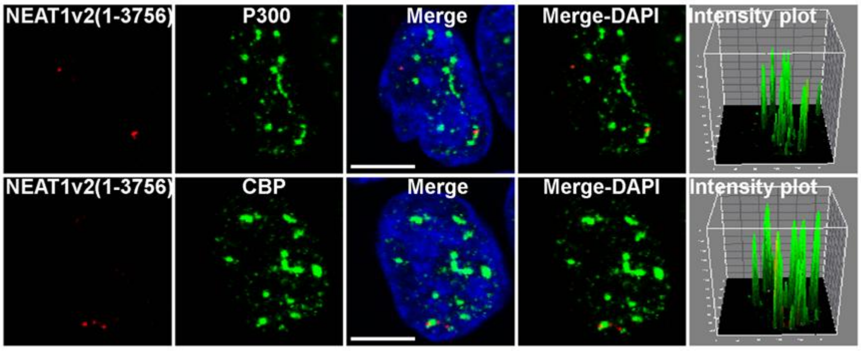

Supplementary Figure 6. The interaction of *NEAT1v1* with P300/CBP complex. HeLa cells infected with HSV-1 for 4 hours were incubated with *NEAT1v2* (1-3756) probe (red), and then incubated with anti-P300 antibodies (green) or anti-CBP antibodies (green). The images were captured with a confocal microscope. The intensity plots for the red and green channels were analyzed by ImageJ software. DAPI (blue) was used to stain the nuclei. Scale bars, 10 μm.

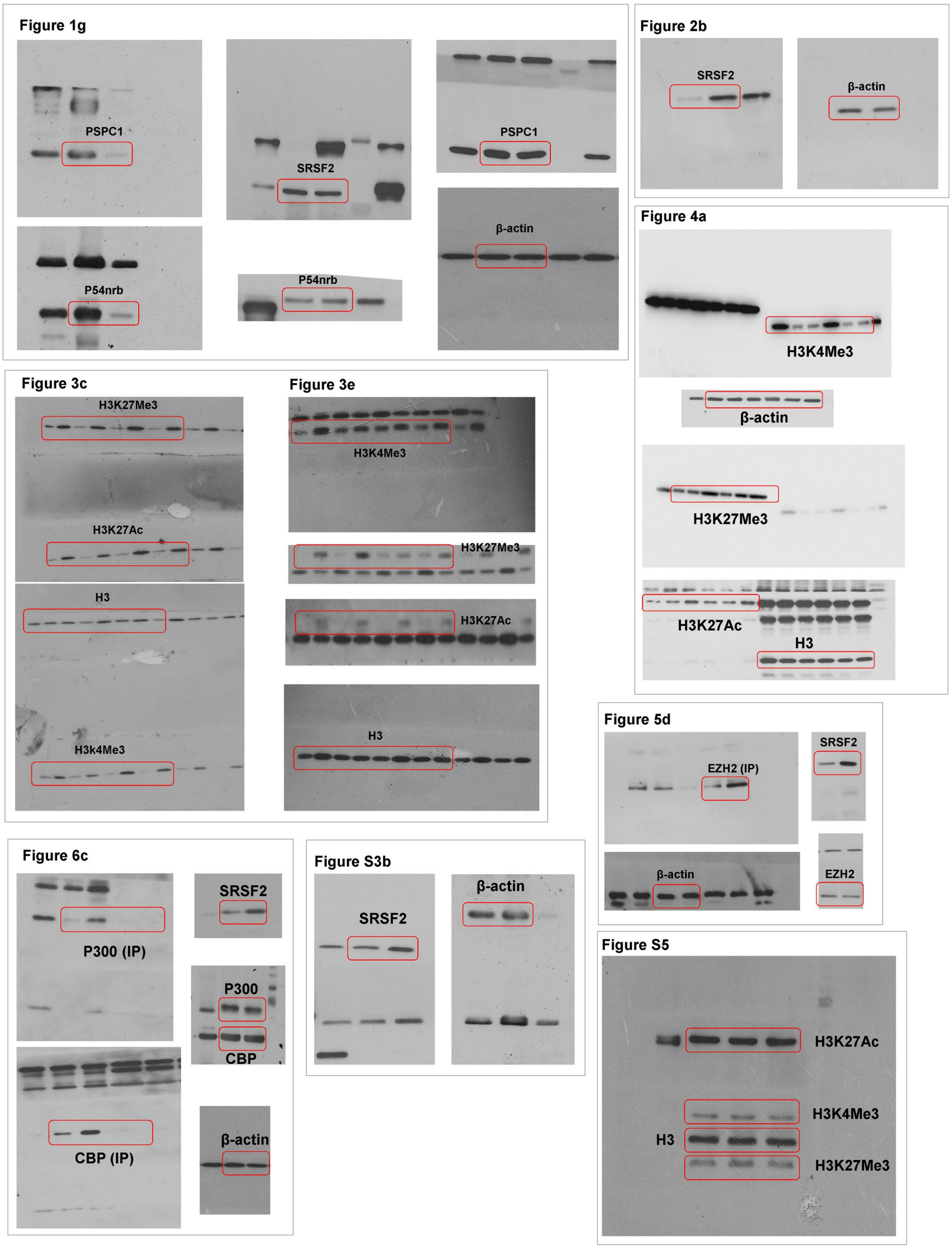

Supplementary Figure 7. Uncropped and unedited blot images.

Supplementary Tables

Supplementary Table 1. Sequences of primers and siRNAs used in this study

SiRNA/primer	Sense Sequence	Anti-sense Sequence
**SiRNA**		
NEAT1-targeting siRNA	CAAACUCUGUACCCAUUAA	UUAAUGGGUACAGAGUUUG
SRSF2-targeting siRNA	GUGAGAAGUUGCUUAGAAA	UUUCUAAGCAACUUCUCAC
Negative control siRNA	UUCUCCGAACGUGUCACGU	ACGUGACACGUUCGGAGAA
**Primers pairs for RIP**		
N1	GCCTTCTTGTGCGTTTCTCG	TCCCAGCGTTTAGCACAACA
N2	GCCATGTAGGAGAGCATGGTA	ACACCCACCTTCATTGGCTT
N3	TCCTCTGAGTGAAGGCATCC	AAAGAGAGGAAAGGCAGTGCAG
N4	TCTCAGAACCCACCTCCTGT	TCAGGGACAAGCAACAACCA
N5	AGCCCATCTCTTGAGCTGTT	CCATAAGTGCTCACTGCCGA
N6	TCCTCTTTGACCACTAACCATGT	AGGTGCTTTTTGCACCAACAAT
N7	TGGAGGCTAGAACACTCCTGT	GAGAGCCATGTTGTGTCCTGA
N8	CCTTGCCCAGGGGATAGTTC	AGAGGCTGAGAGGCAACCTA
N9	GCTTAATGCTGACAAGGCCC	TGCAGGCATAAGCAGAGGAC
N10	TGACCCCAAAGGCAAATGCT	ACATACACAGGTTCTCTGGAAAG
N11	TCTCCTGGCTATTCCAGGCT	GCCGAGGTAGACAGACCAAG
N12	ACAGGTGTTTAGAGGCACAGAA	GGGGTGTCTGCTGACATTCTA
N13	CAAGGGGAGCTGCTGTTTCCT	TGTTACAAGACCCTCCGTGCC
N14	CAGTCTTGCTCTAGCCCCAC	GATGGCATCAGTAGCCTCCC
N15	CTGAGGAAGTGGCCTGTACG	GAGAATCCCTTCCCACTGCC
**Primers pairs for Real Time PCR**		
NEAT1	ACATTGTACACAGCGAGGCA	CATTTGCCTTTGGGGTCAGC
β-actin	TGACGTGGACATCCGCAAAG	CTGGAAGGTGGACAGCGAGG
**Primer pairs for plasmid constructions**		
ICP0-promoter	CGGGGTACCTCTAACGTTACACCCGAGGC	CCGCTCGAGTTCTGTGGTGATGCGGAGAG
ICP4-promoter	CGGGGTACCTCAGACTCCGATGAGAGGGG	CCGCTCGAGCGTCTGACGGTCTGTCTCTG
ICP22-promoter	CGGGGTACCGGTGGGAAAAAGGACAGGGA	CCGCTCGAGGGTGCTTACCCGTGCAAAAA
**Primers pairs for ChIP**		
ICP0-promoter	CCATTGGGGGAATCGTCAC	CTTCTGTGGTGATGCGGAG
TK-promoter	AAACGCGGGCGTATTGGT	ACAATACCGGAAGGAACCCG
ICP0-TSS1	CCGACAGTCTGGTCGCATTT	GGCTCCATGGGGGTCGTAT
ICP0-TSS2	CCATTGGGGGAATCGTCAC	CTTCTGTGGTGATGCGGAG
ICP0-TSS3	GGGCATGCTAATGGGGTTCT	GCAGTGACGATTCCCCCAAT
ICP0-TSS4	CAATGAACCCGCATTGGTCC	AGAACCCCATTAGCATGCCC
TK-TSS1	GGGCGATTGGTCGTAATCCA	CCCAACGGCGACCTGTATAA
TK-TSS2	AAACGCGGGCGTATTGGT	ACAATACCGGAAGGAACCCG
TK-TSS3	GCAGGTAGGTCTTCGGGATG	GGCATCTCTGCCCCTTCTTC
TK-TSS4	AAAAGCCTAGCAGGTCGGAG	CCTCTCTTCTGGCGCCTAAC
